# Cosmetic Use and Its Hidden Risks: An Assessment of Public Awareness and Attitudes in Punjab, India

**DOI:** 10.1111/jocd.71057

**Published:** 2026-07-09

**Authors:** Sweta Kumari, Biplab Pal, Sanjeev Kumar Sahu, Anil Kumar Sah

**Affiliations:** ^1^ School of Pharmaceutical Sciences, Lovely Professional University Phagwara Punjab India; ^2^ Gorkha Ayurved Company Pvt. Ltd. Gorkha Haramtari Nepal

**Keywords:** adverse events, attitude toward cosmetics, consumer safety, cosmetic products, public awareness

## Abstract

**Background:**

The widespread use of cosmetics for aesthetic purposes poses potential health risks due to improper or uninformed usage, leading to skin allergies, irritation, and systemic toxicity from harmful ingredients like heavy metals.

**Objective:**

To assess the knowledge and attitudes of the general population in Punjab, India, regarding cosmetic use and its associated adverse events.

**Methodology:**

A community‐based cross‐sectional survey was conducted from January to March 2024 in Jalandhar, Amritsar, and Ludhiana. A total of 380 participants aged 18–50 years were selected using convenient sampling. Data were collected through a validated 25‐item questionnaire, and descriptive statistics with Bloom's criteria were used for analysis.

**Results:**

Overall, 59.8% of participants had low knowledge, while only 5.8% showed high knowledge levels. Awareness of vitamin C anti‐aging properties (59.5%) and sunscreen use reducing skin care (65.8%) was higher than that of heavy metal toxicity (26.1%). About 41.7% exhibited a positive attitude toward cosmetic safety. Females, younger participants, and those with higher education showed better knowledge and attitudes.

**Conclusion:**

The study highlights a major knowledge gap and moderate attitudes toward cosmetic use. Educational campaigns and joint efforts by health professionals, regulators, and the cosmetic industry are needed to promote safe and informed cosmetic practices.

## 1 Introduction

Cosmetics have been used for thousands of years to enhance appearance, maintain personal hygiene, and improve self‐perception [1]. A cosmetic product is defined as any substance or formulation intended for application to various parts of the human body, such as the skin, hair, nails, lips, and external genital organs, as well as the teeth and oral mucous membranes. The primary purposes of these products are to clean, perfume, alter appearance, correct body odors, or protect and maintain the body in good condition [2]. According to these definitions, cosmetics are classified as products like skin creams, lotions, perfumes, lipsticks, nail polishes, eye and facial makeup, soaps, shampoos, hair dyes, permanent waves, and toothpastes [3]. Although cosmetics are used by both men and women, they are more commonly used by women [4]. Women's more frequent use of cosmetics may be attributed to their emphasis on maintaining their appearance, self‐perception, and beauty [5]. Global marketing is growing in parallel with the rise in cosmetic use. Between 2024 and 2028, global revenue in the “Cosmetics” category of the beauty & personal care industry is expected to increase by 20.5 billion US dollars. After eight consecutive years of growth, it is estimated that the indicator will reach 128.89 billion U.S. dollars, marking a new peak in 2028 [6].

Despite their widespread use to enhance aesthetics and quality of life, cosmetics are increasingly linked to a number of adverse events. These reactions range from localized dermatological complications—such as irritation, erythema, burning sensations, and urticarial rash to systemic reactions [7, 8, 9]. Furthermore, toxicological assessments have revealed that certain cosmetics contain hazardous heavy metals, such as lead and cadmium, which pose severe risks to human health due to their neurotoxic profiles and potential [10]. Additionally, there is growing scientific concern about the content and safety of chemicals used in cosmetics that may act as endocrine‐disrupting chemicals (EDCs), potentially interfering with hormonal [11, 12].

To reduce the cosmetic‐induced adverse events, individuals using cosmetics are strongly advised to follow these safety guidelines: carefully read product labels, adhere to all instructions and warnings, ensure hands are clean prior to application, refrain from sharing makeup, maintain clean and tightly sealed containers, safeguard them from temperature fluctuations, and discard cosmetics if any changes in color or scent occur [13, 14].

Knowledge and attitude studies play an important role in assessing public awareness, health beliefs, and consumer behaviors, thereby identifying critical knowledge gaps and informing targeted educational and public health interventions [15]. Despite the widespread use of cosmetic products and growing concerns regarding their safety, community‐based evidence on public awareness of cosmetic utilization, ingredient‐related risks, and cosmetic‐associated adverse events remains limited in India. Existing studies have predominantly focused on consumer preferences, cosmetic usage patterns, or specific demographic groups, with relatively little attention given to public understanding of cosmetic ingredients, sunscreen efficacy, and potential dermatological adverse effects. Consequently, there is a paucity of data regarding the knowledge and attitudes of the general population toward the safe use of cosmetic products and their associated risks. Therefore, the present study was undertaken to assess the knowledge and attitudes regarding cosmetic utilization and associated adverse events among residents of Punjab, India.

## 2 Materials and Methods

### 2.1 Study Design and Setting

The general population from the Punjab regions of Jalandhar, Amritsar, and Ludhiana was included in this survey. Doaba, Majha, and Malwa are the three geographical divisions of the Punjab. One significant city, such as Jalandhar, Amritsar, or Ludhiana, was chosen from each zone. The survey was conducted between January and March of 2024.

### 2.2 Study Population

Participants' selection was done on the basis of pre‐defined inclusion and exclusion criteria.

#### 2.2.1 Inclusion Criteria

Residents of Jalandhar, Amritsar, and Ludhiana, irrespective of their gender or nationality, were eligible to participate in the study. Individuals who could read and write in Hindi, Punjabi, or English and who regularly used any type of cosmetic product were included. Participants between 18 and 50 years of age who were willing to sign an informed consent form were invited to take part in the study.

#### 2.2.2 Exclusion Criteria

Participants have hearing and sight problems.

### 2.3 Sample Size

The minimum required sample size was calculated using the single proportion formula *n* = *Z* ^2^ * *P*(1 − *P*)/*d* ^2^, where *n* is the target sample size, *Z* = 1.96, for 95% confidence interval, *d* = 0.05% (margin of error) and *p* = 62.6%, obtained from a prior study [16]. The sample size was found to be 359. To increase the power of the study, a total of 380 participants were enrolled.

### 2.4 Data Collection

By referencing previously published articles, a structured, self‐administered questionnaire was developed and subsequently modified as required [8, 16]. Closed‐ended questions were included in the questionnaire. The questionnaire was first developed in English and later translated into Punjabi and Hindi. Punjabi is the regional language of Punjab, whereas Hindi is the official language of India. The 25‐item survey was roughly divided into three sections; Section A included participant's sociodemographic information like age, gender, marital status, place of residence, family monthly income, occupation, and level of education (Q1–Q7), section B related to knowledge regarding cosmetic utilization and adverse events such as what is the first step of facial skincare, which is the appropriate skin type for using cosmetic powders, Which vitamin is commonly found in skincare products and is known for antiaging properties etc. (Q8–Q18) and section C included questions related to attitude regarding cosmetics utilization and adverse events such as use of cosmetics inappropriately can lead to wrinkles, skin darkening, and rashes, quality matters more than price in cosmetics etc. (Q19–Q25). A 5‐point Likert scale was used to identify the attitude regarding cosmetics utilization and adverse events.

### 2.5 Validation

Five experts from diverse fields, including physicians, biostatisticians, and doctoral‐level academic pharmacists, assessed the content validity of the questionnaire. All experts had prior experience in questionnaire‐based research. Content validity was evaluated using the Lawshe method [17]. Item‐level CVIs (I‐CVIs) were calculated and then averaged to obtain the scale‐level content validity index (S‐CVI/Ave), which was found to be 0.91, indicating excellent content validity. For face validity, the questionnaire was administered to 20 community members; however, their responses were not included in the final analysis. Reliability of the scale was assessed using SPSS version 16 by calculating Cronbach's alpha. The 25‐item scale demonstrated acceptable internal consistency, with a Cronbach's alpha value of 0.73.

### 2.6 Administration of Questionnaire

The questionnaire was distributed to the public across various locations, including schools, colleges, shopping malls, medical stores, general stores, markets, hospitals, and parks. Study participants were selected using a convenient sampling method. The purpose of the study was clearly explained to all participants, and informed consent was obtained prior to completing the questionnaire. For illiterate participants, the investigator conducted a face‐to‐face interview to ensure accurate responses. All questionnaires were reviewed for completeness, and only fully completed forms were included in the final analysis.

### 2.7 Ethical Consideration

Approval of the study protocol was obtained from the Institutional Ethics Committee and all the participants provided written informed consent before taking part in the study.

### 2.8 Data Analysis

The data were processed with SPSS (Statistical Packages for Social Sciences) version 16. Descriptive statistics (number and percentage) were used to summarize demographic information, knowledge and attitude about cosmetic use, and adverse occurrences. Participants' knowledge was assessed by assigning a score of 1 for correct answers and 0 for incorrect, and each participant's overall knowledge scores were calculated. The attitude score was computed by aggregating the ratings (ranging from 1 to 5) provided by each participant for every question. Bloom's cut‐off point was used to find out the high knowledge score and positive attitude of the participants [18, 19]. Descriptive statistics (number and percentage) were used to summarize demographic characteristics, knowledge and attitude of participants regarding cosmetic utilization and associated adverse events.

## 3 Result

### 3.1 Sociodemographic Characteristics

This study enrolled a total of 380 participants, with 60.8% participant within the 18–28 age group. The majority of participants were female (52.4%), and a significant proportion were unmarried (57.1%). Additionally, 30.5% reported a monthly income > 60 000, while 50% held a graduate or higher degree, and 43.4% have private jobs. Sociodemographic characteristics of the participants is given in Table 1.

**TABLE 1:** Sociodemographic characteristics of the participants.

Variables	*N* (%)
*Age*	
18–28	231 (60.8)
29–39	107 (28.2)
40–50	42 (11.1)
*Gender*	
Male	181 (47.6)
Female	199 (52.4)
Transgender	0 (0)
*Marital status*	
Single	217 (57.1)
Married	159 (41.8)
Divorced	3 (0.8)
Widow	1 (0.3)
*Residence*	
Urban	278 (73.2)
Rural	102 (26.8)
*Family monthly income (Rs)*	
< 10 000	34 (8.9)
10 001–20 000	39 (10.3)
20 001–40 000	95 (25)
40 001–60 000	96 (25.3)
> 60 000	116 (30.5)
*Educational status*	
Illiterate	6 (1.6)
Primary	23 (6.1)
10th	29 (7.6)
12th	132 (34.7)
Graduate & higher	190 (50)
*Occupation*	
Student	108 (28.4)
Unemployed	9 (2.4)
House maker	37 (9.7)
Govt. employee	18 (4.7)
Private job	165 (43.4)
Business	43 (11.3)

### 3.2 Knowledge of the Participants Regarding Cosmetics Utilization and Adverse Events

The score (mean ± SD) of participants' knowledge about cosmetic utilization and adverse events was 4.72 ± 2.41 (ranging 0–11). The majority of participants, 51.8%, were aware that cleanser milk is the initial step in facial skincare, whereas 47.4% possessed the correct understanding that cosmetic powder is most suitable for oily skin types. Likewise, 59.5% accurately identified vitamin C as a common ingredient in skincare products known for its anti‐aging properties. Additionally, 60% correctly stated that sunscreen should be applied 15–30 min prior to sun exposure. Among participants, 43.4% correctly answered that petroleum jelly is the main ingredient in most lip balms that help moisturize the lips. Moreover, 35% knew deep mask is an appropriate conditioner to use after hair dyeing. However, 43.7% answered that dandruff is the most common side effect of hair dyes. Only 26% of participants knew that heavy metals, when present in elevated levels in cosmetics, can lead to toxicities with prolonged use. More than half of the participants (65.8%) answered that sunscreen helps in reducing the risk of skin cancer. Very few (11.1%) participants knew that fragrances in perfumes are the most common cause of cosmetic‐induced skin allergy. Similarly, only 28.7% were correct that oil‐based cosmetics are most likely to have side effects on the skin. Further details regarding the participants' knowledge concerning cosmetic utilization and adverse events are provided in the accompanying Table 2.

**TABLE 2:** Distribution of participant's knowledge regarding cosmetics utilization and adverse events.

Questions	Correct	Incorrect/do not know	Correct answer
	*N* (%)	*N* (%)	
What is the first step in facial skincare?	197 (51.8)	183 (48.2)	Cleanser milk
Which is the appropriate skin type for using cosmetic powders?	180 (47.4)	200 (52.6)	Oily skin
Which vitamin is commonly found in skincare products and is known for antiaging properties?	226 (59.5)	154 (40.5)	Vitamin C
When should sunscreen be applied before sun exposure?	228 (60.0)	152 (40.0)	15–30 min before sun exposure
What is the main ingredients in most lip balm that help moisturize the lip?	165 (43.4)	215 (56.6)	Petroleum jelly
What is an appropriate conditioner to use after hair dyeing?	133 (35.0)	247 (65.0)	Deep mask
What is the most common side effect of hair dyes?	166 (43.7)	214 (56.3)	Dandruff
Which metal, when present in elevated levels in cosmetics can lead to toxicities with prolonged use	99 (26.1)	281 (73.9)	Heavy metal
Which cosmetics help in reducing the risk of skin cancer?	250 (65.8)	130 (34.2)	Sunscreen
What is the most common cause of cosmetic‐induced skin allergy?	42 (11.1)	338 (88.9)	Fragrances in perfumes
Which type of cosmetics is most likely to have side effects on the skin?	109 (28.7)	271 (71.3)	Oil based cosmetics

### 3.3 Attitude of the Participants Regarding Cosmetics Utilization and Adverse Events

The participants' attitudes toward cosmetic utilization and adverse events were assessed, yielding a mean (±SD) score of 26.09 ± 3.91. Notably, less than half of the participants (42.6%) strongly agreed that inappropriate cosmetic use could result in adverse effects such as wrinkles, skin darkening, and rashes. Conversely, a majority (54.4%) strongly favored prioritizing quality over price when selecting cosmetics. However, confidence in the safety of cosmetics made with organic herbal ingredients was less prevalent, with only 25.3% of participants strongly agreeing on their safety and health benefits. Additionally, a minority (13.4%) held the belief that tattoos could potentially lead to cancer. Concerns about adverse effects extended to hair care products, as 23.2% strongly agreed that chemical hair dyes and bleaching could cause gray hair. On preventive measures, only 12.1% acknowledged the importance of beginning sunscreen use at a young age. Conversely, a notable percentage (31.3%) believed that water is commonly used to remove makeup. Details of attitudes of the participants regarding cosmetics utilization and adverse events are discussed in Table 3.

**TABLE 3:** Distribution of participants' attitude regarding cosmetics utilization and adverse events.

Questions	Strongly agree	Agree	No idea	Disagree	Strongly disagree
Use of cosmetics inappropriately can lead to wrinkles, skin darkening, and rashes	162 (42.6%)	142 (37.4%)	37 (9.7%)	20 (5.3%)	19 (5%)
Quality matters more than price in cosmetics	207 (54.4%)	118 (31.1%)	33 (8.7%)	6 (1.6%)	16 (4.2%)
Are cosmetics made with organic herbal ingredients safer and healthier?	96 (25.3%)	166 (43.7%)	71 (18.7%)	35 (9.2%)	12 (3.1%)
Can a tattoo cause cancer?	51 (13.4%)	137 (36.1%)	105 (27.6%)	59 (15.5%)	28 (7.4%)
Gray hair could be a side effect of chemical hair dyes and bleaching	88 (23.2%)	157 (41.3%)	91 (23.9%)	32 (8.4%)	12 (3.2%)
It is important to start using sunscreen at a young age	46 (12.1%)	133 (35%)	108 (28.4%)	70 (18.4%)	23 (6.1%)
Usually, water is used to remove makeup	119 (31.3%)	127 (33.4%)	39 (10.3%)	51 (13.4%)	44 (11.6%)

### 3.4 Comparison of Knowledge and Attitude Scores Among Different Demographic Variables

Only 5.8% participants had high knowledge, and 41.7% had a positive attitude. Details are given in Table 4. Younger generations between the ages of 18 and 28 were found to have high levels of knowledge and positive attitudes. Additionally, both knowledge and attitudes were higher among females. Similarly, unmarried individuals, students, and those with higher educational qualifications had higher knowledge scores. The details of the comparison of knowledge and attitude among different demographic variables are given in Tables 5 and 6.

**TABLE 4:** Categorization of knowledge and attitude scores based on Bloom's criteria.

	Category	Score	*N* (%)
Knowledge	High	9–11 (80%–100%)	22 (5.8)
	Moderate	6–8 (60%–79%)	130 (34.1)
	Low	< 6 (< 60%)	228 (59.8)
Total			380 (100)
Attitude	Positive	28–35 (80%–100%)	159 (41.7)
	Neutral	21–27 (60%–79%)	193 (50.7)
	Negative	< 20 (< 60%)	28 (7.3)
Total			380 (100)

**TABLE 5:** Comparison of knowledge among different demographic variables.

Variables	High knowledge (*N* = 22)	Moderate knowledge (*N* = 130)	Low knowledge (*N* = 228)	Total (*N* = 380)
*Age*				
18–28	14 (63.6%)	91 (70%)	126 (55.2%)	231 (60.7%)
29–39	7 (31.8%)	30 (23%)	70 (30.7%)	107 (28.1%)
40–50	1 (4.5%)	9 (6.9%)	32 (14%)	42 (11%)
*Gender*				
Male	10 (45.4%)	45 (34.6%)	126 (55.2%)	181 (47.6%)
Female	12 (54.5%)	85 (65.3%)	102 (44.7%)	199 (52.3%)
Transgender	0 (0%)	0 (0%)	0 (0%)	0 (0%)
*Marital status*				
Single	17 (77.2%)	92 (70.7%)	108 (47.3%)	217 (57.1%)
Married	5 (22.7%)	36 (27.6%)	118 (51.7%)	159 (41.8%)
Divorced	0 (0%)	1 (0.7%)	2 (0.8%)	3 (0.7%)
Widow	0 (0%)	1 (0.7%)	0 (0%)	1 (0.2%)
*Residence*				
Urban	17 (77.2%)	100 (76.9%)	161 (70.6%)	278 (73.1%)
Rural	5 (22.7%)	30 (23%)	67 (29.3%)	102 (26.8%)
*Family monthly income (Rs)*				
< 10 000	0 (0%)	9 (6.9%)	25 (10.9%)	34 (8.9%)
10 001–20 000	0 (0%)	12 (9.2%)	27 (11.8%)	39 (10.2%)
20 001–40 000	6 (27.2%)	30 (23%)	59 (25.8%)	95 (25%)
40 001–60 000	2 (9%)	28 (21.5%)	66 (28.9%)	96 (25.2%)
> 60 000	14 (63.6%)	51 (39.2%)	51 (22.3%)	116 (30.5%)
*Educational status*				
Illiterate	1 (4.5%)	1 (0.7%)	4 (1.7%)	6 (1.5%)
Primary	4 (18.1%)	3 (2.3%)	16 (7%)	23 (6%)
10th	2 (9%)	9 (6.9%)	18 (7.8%)	29 (7.6%)
12th	5 (22.7%)	42 (32.3%)	85 (37.2%)	132 (34.7%)
Graduate & higher	10 (45.4%)	75 (57.6%)	105 (46%)	190 (50%)
*Occupation*				
Student	14 (63.6%)	46 (35.3%)	48 (21%)	108 (28.4%)
Unemployed	0 (0%)	1 (0.7%)	8 (3.5%)	9 (2.3%)
House maker	1 (4.5%)	7 (5.3%)	29 (12.7%)	37 (9.7%)
Govt. employee	0 (0%)	5 (3.8%)	13 (5.7%)	18 (4.7%)
Private job	7 (31.8%)	68 (52.3%)	90 (39.4%)	165 (43.4%)
Business	0 (0%)	3 (2.3%)	40 (17.5%)	43 (11.3%)

**TABLE 6:** Comparison of attitude among different demographic variables.

Variables	High attitude (*N* = 159)	Moderate attitude (*N* = 193)	Low attitude (*N* = 28)	Total (*N* = 380)
*Age*				
18–28	101 (63.5%)	111 (57.5%)	19 (67.8%)	231 (60.8%)
29–39	38 (23.9%)	63 (32.6%)	6 (21.4%)	107 (28.1%)
40–50	20 (12.6%)	19 (9.8%)	3 (10.7%)	42 (11.1%)
*Gender*				
Male	67 (42.1%)	98 (50.8%)	16 (57.1%)	181 (47.6%)
Female	92 (57.9%)	95 (49.2%)	12 (42.9%)	199 (52.4%)
Transgender	0 (0%)	0 (0%)	0 (0%)	0 (0%)
*Marital status*				
Single	88 (55.3%)	111 (57.5%)	18 (64.3%)	217 (57.1%)
Married	69 (43.4%)	80 (41.5%)	10 (35.7%)	159 (41.8%)
Divorced	1 (0.6%)	2 (1%)	0 (0%)	3 (0.8%)
Widow	1 (0.6%)	0 (0%)	0 (0%)	1 (0.3%)
*Residence*				
Urban	116 (73%)	140 (72.5%)	22 (78.6%)	278 (73.2%)
Rural	43 (27%)	53 (27.5%)	6 (21.4%)	102 (26.8%)
*Family monthly income (Rs)*				
< 10 000	8 (5%)	21 (10.8%)	5 (17.8%)	34 (8.9%)
10 001–20 000	17 (10.6%)	16 (8.2%)	6 (21.4%)	39 (10.2%)
20 001–40 000	38 (23.8%)	47 (24.3%)	10 (35.7%)	95 (25%)
40 001–60 000	46 (28.9%)	49 (25.3%)	1 (3.5%)	96 (25.2%)
> 60 000	50 (31.4%)	60 (31%)	6 (21.4%)	116 (30.5%)
*Educational status*				
Illiterate	3 (1.9%)	3 (1.6%)	0 (0%)	6 (1.6%)
Primary	7 (4.4%)	14 (7.3%)	2 (7.1%)	23 (6.1%)
10th	16 (10.1%)	13 (6.7%)	0 (0%)	29 (7.6%)
12th	46 (28.9%)	75 (38.9%)	11 (39.3%)	132 (34.7%)
Graduate & higher	87 (54.7%)	88 (45.6%)	15 (53.6%)	190 (50%)
*Occupation*				
Student	52 (32.7%)	52 (26.9%)	4 (14.3%)	108 (28.4%)
Unemployed	5 (3.1%)	2 (1%)	2 (7.1%)	9 (2.4%)
House maker	20 (12.6%)	16 (8.3%)	1 (3.6%)	37 (9.7%)
Govt. employee	11 (6.9%)	6 (3.1%)	1 (3.6%)	18 (4.7%)
Private job	55 (34.6%)	92 (47.7%)	18 (64.3%)	165 (43.4%)
Business	16 (10.1%)	25 (12.9%)	2 (7.1%)	43 (11.3%)

## 4 Discussion

In the present study, only 22 (5.8%) participants had high knowledge regarding cosmetic use and associated adverse events, indicating insufficient awareness and understanding among the majority of respondents. These results show that despite the widespread use of cosmetics, the general public still lacks sufficient knowledge about their safe usage, components, and possible side effects. This result is consistent with research conducted in Saudi Arabia [20] and South Africa [21], where women showed limited awareness regarding cosmetic safety. The relatively low level of knowledge observed in our study may be due to a number of factors. Cosmetics are frequently seen as everyday consumer goods rather than something that could have negative health impacts. Customers tend to pay little attention to product contents, usage guidelines, and safety warnings. Additionally, there are a few organized public awareness efforts in India that concentrate on cosmetic safety and cosmetovigilance. For information about cosmetics, consumers usually rely on friends, family, commercials, and social media influencers. These sources generally highlight the advantages and beauty results of the products rather than any possible health risks or negative effects.

In contrast, most Korean [22] and Lebanese women [16], showed a high level of awareness regarding the usage of cosmetics. These differences may be explained by variations in educational attainment, socioeconomic status, cultural perspectives on skincare, increased consumer involvement with cosmetic products, and improved access to cosmetic‐related information through medical professionals, media campaigns, and regulatory initiatives. Additionally, Lebanon has reported relatively high cosmetic use among women, which may contribute to greater familiarity with cosmetic products and their proper use, whereas South Korea has a highly developed beauty and skincare industry with strong consumer awareness regarding cosmetic ingredients and product safety. The variance in results across nations may also be explained by variations in study populations, survey methodologies, and healthcare awareness initiatives.

Approximately half of the participants did not correctly identify the first step in facial skincare as cleanser milk, which is contrary to the findings in Lebanese women [16]. However, the relatively lower recognition of oily skin as the appropriate type for using cosmetic powders suggests a potential area for improvement in consumer education, aligning with the findings of Chahine et al. [16]. There is a lack of knowledge among consumers concerning skincare, which may be due to the informal and often traditional ways cosmetic information is passed on from family members or peers or learned via social media rather than evidence‐based sources.

Additionally, while a substantial portion recognized vitamin C for its anti‐aging properties, there remains scope for enhancing awareness, especially considering its prevalence in skincare products [23] The considerably increased awareness of vitamin C‐containing skincare products may be explained by their increasing popularity and widespread marketing activities.

The significant proportion of participants aware of the optimal timing for sunscreen application is encouraging, and may reflect increasing public awareness of sun protection through social media, healthcare professionals, and cosmetic marketing campaigns. This finding is similar to Chahine et al. [16] while contrasting with findings from a Malaysian study [24] and Wang and Dusza [25], possibly due to differences in study populations, educational levels, and exposure to skin health information.

However, the relatively lower recognition of petroleum jelly as a primary lip balm ingredient underscores the need for increased emphasis on awareness of cosmetic ingredients among users. Similarly, the limited awareness of deep masks as a post‐hair dyeing conditioner suggests gaps in understanding hair care routines. Moreover, the low awareness of the potential toxicity associated with heavy metals in cosmetics emphasizes the need for increased awareness of ingredient safety and the misconception that commercially available products are inherently safe [26]. A considerable number of participants recognized sunscreen for its role in reducing skin cancer risk, aligning with the findings from a study conducted by Wang and Dusza [25]. This may be due to increasing exposure to sun protection messages through healthcare professionals, media campaigns, and cosmetic advertisements. However, the low awareness of fragrances in perfumes as a common cause of cosmetic‐induced skin allergies and oil‐based cosmetics as likely to have adverse effects on the skin highlights the need for targeted educational interventions focusing on cosmetic ingredients and their potential health risks [27].

A significant number of participants in the study agreed that inappropriate use of cosmetic products could lead to adverse effects, emphasizing the importance of proper cosmetic use to avoid skin issues such as wrinkles, darkening, and rashes. Similar observations were reported in studies conducted in Lebanon [16] and India [28], indicating a growing awareness among consumers about the significance of skincare practices. Additionally, the majority of participants expressed a preference for quality overpriced in cosmetics, suggesting a general inclination toward prioritizing product effectiveness and safety over affordability. This is consistent with findings in Nepal [29], where consumers considered quality before price while purchasing cosmetics. Moreover, most participants asserted to trust cosmetics made with organic herbal ingredients more than chemical‐based ones, which is supported by results from studies conducted in the UK [30], Malaysia [31], and India [32]. This may be influenced by marketing strategies promoting natural products as healthier alternatives. However, this perception may not always reflect actual product safety, emphasizing the need for consumer education regarding evidence‐based evaluation of cosmetic products.

Similarly, there was a common perception among participants regarding the relationship between chemical hair dyes/bleaching and gray hair. Most participants had the belief that water could effectively be used for makeup removal, whereas opinions were divided on starting sunscreen use at a young age, highlighting the necessity for targeted education initiatives to promote proper makeup removal techniques and sunscreen practices. Furthermore, mixed responses regarding tattoos' potential to cause cancer suggested a lack of clarity among participants, emphasizing the importance of disseminating accurate information on tattoo safety. These findings collectively highlight the need for comprehensive educational initiatives aimed at improving consumer understanding of cosmetic safety, appropriate product use, and potential adverse effects.

## 5 Limitation

This study has certain limitations. As a cross‐sectional survey, it only reflects knowledge, attitudes, and adverse events at one point in time and cannot establish causality. The data are self‐reported, which may lead to recall or reporting bias. Since the study was conducted in selected communities of Punjab, the findings may not be generalizable to other regions. Adverse events were not clinically verified, limiting the accuracy of reported reactions. The study did not analyze cosmetic ingredients, preventing correlation with specific products. Additionally, seasonal variations in cosmetic use were not captured.

## 6 Conclusion

These results highlight the necessity of targeted educational campaigns and consumer awareness campaigns to raise awareness of ingredient composition, potential side effects, and cosmetic safety. Enhancing cosmetovigilance practice and disseminating evidence‐based information can enable consumers to make knowledgeable choices about the use of cosmetics. To raise awareness, promote adverse event reporting, and promote ethical cosmetic practices, cooperation between healthcare practitioners, regulatory bodies, cosmetic producers, and public health organizations is crucial. These actions might enhance consumer safety and make the cosmetics sector more dependable and transparent.

## Author Contributions

S.K. original manuscript writing, B.P. data curation and data analysis, S.K.S. conceptualization, supervision and visualization, A.K.S. reviewing and editing.

## Funding

The authors have nothing to report.

## Ethics Statement

Study protocol was approved by the Institutional Ethics Committee of Lovely Professional University (Ref. No.: LPU/IEC‐LPU/2023/1/25).

## Consent

All participants provided written informed consent before participating in the study.

## Conflicts of Interest

The authors declare no conflicts of interest.

## Data Availability

The data that support the findings of this study are available from the corresponding author upon reasonable request.
