# Correction to “Cosmetic Use and Its Hidden Risks: An Assessment of Public Awareness and Attitudes in Punjab, India”

**DOI:** 10.1111/jocd.71226

**Published:** 2026-10-01

S. Kumari , B. Pal , S. K. Sahu , and A. K. Sah , “Cosmetic Use and Its Hidden Risks: An Assessment of Public Awareness and Attitudes in Punjab, India,” Journal of Cosmetic Dermatology 25, no. 7 (2026): e71057, 10.1111/jocd.71057.

The first affiliation was incorrect and has been corrected to “School of Pharmaceutical Sciences, Lovely Professional University, Phagwara, Punjab, India” to accurately reflect the institutional affiliation associated with the work.

We apologize for this error.

